# A method to determine a personalized set of online exercises for improving the positive mental health of a caregiver of a chronically ill patient

**DOI:** 10.1186/s12911-021-01445-6

**Published:** 2021-02-25

**Authors:** Maria Ferré-Bergadà, Aida Valls, Laia Raigal-Aran, Jael Lorca-Cabrera, Núria Albacar-Riobóo, Teresa Lluch-Canut, Carme Ferré-Grau

**Affiliations:** 1 Dept. Enginyeria Informàtica i Matemàtiques, Universitat Rovira i Virgili, Avda. Països Catalans, 26, 43007 Tarragona, Catalonia Spain; 2 Nursing Department, Universitat Rovira i Virgili, Avinguda Catalunya 35, 43002 Tarragona, Catalonia Spain; 3 Nursing Department, Universitat de Barcelona, Carrer de La Feixa Llarga S/N, 08907 L’Hospitalet de Llobregat, Barcelona Spain

**Keywords:** Caregivers, Positive mental health, Mobile health, Utility measurement, Personalization

## Abstract

**Background:**

Taking care of chronic or long-term patients at home is an arduous task. Non-professional caregivers suffer the consequences of doing so, especially in terms of their mental health. Performing some simple activities through a mobile phone app may improve their mindset and consequently increase their positivity. However, each caregiver may need support in different aspects of positive mental health. In this paper, a method is defined to calculate the utility of a set of activities for a particular caregiver in order to personalize the intervention plan proposed in the app.

**Methods:**

Based on the caregivers’ answers to a questionnaire, a modular averaging method is used to calculate the personal level of competence in each positive mental health factor. A reward-penalty scoring procedure then assigns an overall impact value to each activity. Finally, the app ranks the activities using this impact value.

**Results:**

The results of this new personalization method are provided based on a pilot test conducted on 111 caregivers. The results indicate that a conjunctive average is appropriate at the first stage and that reward should be greater than penalty in the second stage.

**Conclusions:**

The method presented is able to personalize the intervention plan by determining the best order of carrying out the activities for each caregiver, with the aim of avoiding a high level of deterioration in any factor.

## Background

Continuous long-term care of dependent people may create stress for the caregiver. This caregiving situation often involves a modification of the usual lifestyle of the non-professional caregiver, too. In that setting, caregivers may feel alone, tired and dejected, which affects their personal quality of life. Therefore, non-professional caregivers also need assistance and help in carrying out the hard work of taking care of these patients. According to Bauer and Sousa-Poza [1], caregivers’ feelings are strongly related to their psychological health, which consequently affects their physical health. This is a consequence of the caregiver burden, a concept defined by the North American Nursing Diagnosis Association [2].

The World Health Organization [3] indicates that mental and physical health are highly interrelated because one depends on the other. The concept of mental health, in its wider sense and with absence of any disorders, is defined as a state of emotional well-being which facilitates having a positive lifestyle. According to Lluch-Canut [4] it is called positive mental health (PMH).

Faronbi et al. [5] highlighted the need to develop specific interventions to support and empower caregivers in taking care of themselves. As a matter of fact, healthcare professionals are increasing their concern about this issue and developing proposals to improve caregivers’ self-care.

In a previous paper by the authors, Ferré-Grau et al. [6] created an intervention plan using problem solving-techniques as a strategy to prevent anxiety and depression. The level of positive mental health (PMH) is assessed through a questionnaire defined and validated by Lluch-Canut [4], Mantas et al. [7], Roldán-Merino et al. [8] and Puig-Llobet et al. [9]. This questionnaire includes 39 items unevenly distributed across the six factors that define the concept of PMH: Factor 1—Personal satisfaction (eight items), Factor 2—Prosocial attitude (five items), Factor 3—Self-control (five items), Factor 4—Autonomy (five items), Factor 5—Problem-solving and self-actualization (nine items) and Factor 6—Interpersonal relationship skills (seven items). The items take the form of positive or negative statements that are answered on a scale from 1 to 4, according to how frequently they occur: always or almost always, quite often, sometimes, rarely or never. The questionnaire provides a global score for PMH (sum of the item scores) as well as specific scores for each factor. It has been validated by different studies, achieving an alpha between 0.89 and 0.90 and a test–retest correlation of 0.85. Based on this questionnaire validation, a decalogue of recommendations to promote PMH was developed by Lluch-Canut [10] and used in papers by Lluch-Canut [11]. These 10 recommendations are challenges that can be accomplished by providing the caregivers with appropriate exercises that focus on these aspects. The daily organization of these home caregivers makes it difficult for them to do these activities in a medical centre. For this reason, using mobile phones in such an intervention plan is very suitable. Therefore, a smartphone app was designed, built and validated by the authors Ferré-Grau et al. [12]. This app proposes caregivers a set of exercises designed to improve different aspects of their mental health.

This paper continues this work in order to improve the app by including a procedure to decide the best order of presenting the exercises to each caregiver, based on the needs of strengthening each person’s different aspects of mental health. The goal is to define a methodology that will allow the app to automatically select and rank a set of exercises for each caregiver that will properly prevent the deterioration of some mental health aspects. To achieve that goal, once a person’s level of PMH is evaluated, it is necessary to define a reasonable, flexible and easy-to-use mechanism to improve it. The tools used to calculate the impact of each exercise and rank them are based on mathematical operations and artificial intelligence techniques for personalization. The methodology designed and explained in section “Methods” is the main contribution of the paper. The results indicate that performing the exercises in the calculated order can improve the caregivers’ positive mental health in a shorter time.

## Methods

In this section, we present the starting point of this paper, which is the mobile application that was developed to improve the caregivers’ positive mental health by Ferré-Grau et al. [12]. Next, the contribution of the paper is explained, which involves a new methodology designed to personalize the order of presentation of the different exercises to each caregiver.

### The app for PMH improvement at home

Starting from the theory of PMH described by the World Health Organization [2] and the decalogue of recommendations to promote PMH defined by Lluch-Canut [10], a mobile phone application (app) was implemented. The multi-factor model comprises six factors: Personal Satisfaction (F1), Prosocial Attitude (F2), Self-control (F3), Autonomy (F4), Problem-solving and Self-actualization (F5) and Interpersonal Relationship Skills (F6). The goal is to know the level of satisfaction of these 6 factors for each caregiver. A scale of 4 values was defined, from 0 (missing) to 3 (maximum presence of the factor). A multidisciplinary team, including home care experts and psychologists, designed a set of 39 questions to assess the current level of satisfaction with each of the factors described by Lluch-Canut et al. [4].

**Fig. 1:**
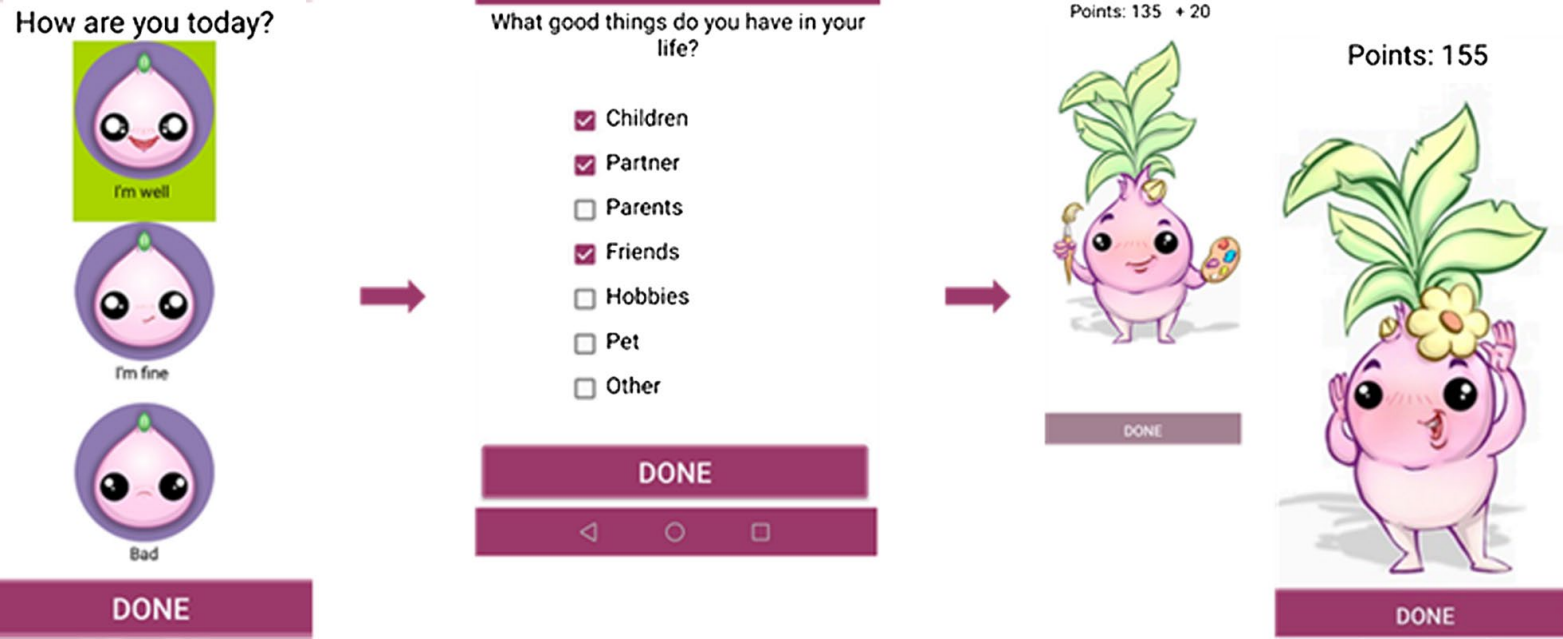
App interface examples: **a** first, every day, the app asks the caregiver how she/he feels **b** next, the application presents the corresponding activity and, finally, **c** the application shows the evolution of the avatar and the points that the caregiver won

This app has a fixed plan that includes 20 exercises that all caregivers must do for 4 weeks. In this way, the app proposes every day (from Monday to Friday) an activity to promote some of the PMH factors. To facilitate the user engagement with the training plan, the app uses gamification techniques, including an avatar and earning points when the activities are completed. Motivational messages are also displayed when appropriate. Improvement in factor levels can be measured after the 4 weeks are completed, when the caregiver repeats the initial test. The application interface has been carefully designed to appeal to the caregiver (Fig. 1). The details of the interface design, the intervention plan and the validation study of this smartphone app can be found in Ferré-Grau et al. [13].

A limitation of this first version of the app is that the exercises are presented to all caregivers in the same order. In effect, depending on the needs of each caregiver, some exercises should be done before others. To avoid this limitation, this paper presents a methodology to adapt the app to each user.

### Methodology to personalize the ranking of the training exercises

To determine the best order of presenting the different training activities to each user, we can use automatic decision-making support techniques. These methods are well studied and applied in fields like artificial intelligence, operational research and economics. In this paper, we will follow a well-known approach that originated in the 1970s known as multi-attribute utility theory (MAUT), Keeney et al. [14]. MAUT methods are focused on evaluating the performance of a set of options in terms of suitability for a user (i.e., utility) based on specific criteria.

In this work, the options to be evaluated are the set of activities available in the app. This set is fixed and expressed as $A = \left\{ {a_{1} , \ldots ,a_{n} } \right\}$*.* To evaluate the utility of the activities, we will use the information provided by the answers to the pre-test questionnaire, as these answers give an evidence of the strengths and needs of a caregiver before starting to use the intervention plan proposed by the app.

**Fig. 2:**
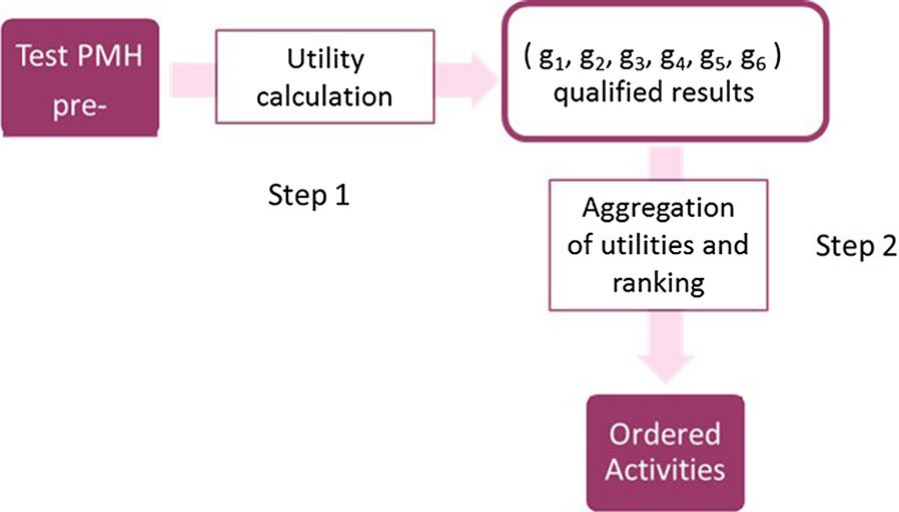
Flowchart of the of the caregiver’s actions revisited

Each caregiver must perform all the activities, but the order in which they are proposed is important to avoid the deterioration of the weak PMH factors, while keeping the others at a good level. To achieve this user-centered proposal of exercises, the system performs two steps before starting to send activities to the user (Fig. 2):

- STEP 1: the answers of the caregiver for each factor are aggregated and together with the impacts of the activity, a marginal utility score is calculated for each factor and activity.
- STEP 2: an overall utility score is calculated for each activity, which is then used to establish the order.

### Calculation of the marginal utility scores for a given caregiver

In the proposed methodology, the first step assigns a negative, zero or positive utility score to each factor of each activity. The utility score is obtained using the designed algorithm explained in this section, using as input the answers given by the caregiver on a survey carried out before starting the intervention plan.

First, the caregiver’s level of competence in each of the *p* PMH factors $F = \left\{ {f_{1} , \ldots ,f_{p} } \right\}$ is estimated based on the answers to the survey. For each caregiver *c*, there is a vector with the current achievement levels of PMH: $L\left( c \right) = \left\{ {l_{1} \left( c \right), \ldots ,l_{p} \left( c \right)} \right\}$. The levels are expressed as a numerical score in the range of 0 to 3. The higher the score, the higher the level of achievement of this positive mental health factor, which means that the caregiver does not need to improve it. The critical factors are the ones with the lowest scores.

The survey has *m* questions; each of them intends to elicit information on a subset of the PMH factors. As explained before, each caregiver, *c*, has answered to every question, *q*, with a numerical score $s^{c} \left( q \right) \in \left[ {0 \ldots 3} \right]$. We assume each score gives some evidence about the level of satisfaction of all the factors related to this question. This relationship is represented with a Boolean vector $R\left( q \right) = \left\{ {r_{1} \left( q \right), \ldots ,r_{p} \left( q \right)} \right\}$, where $r_{j}$ are defined in Eq. 1.

1 $$r_{j} \left( q \right) = \left\{ {\begin{array}{*{20}l} 0 \hfill & {q\,does\,not\,provide\,evidence\,about\,f_{j} } \hfill \\ 1 \hfill & {q\,provides\,evidence\,about\,f_{j} } \hfill \\ \end{array} } \right.$$

To calculate a caregiver *c*’s overall level of competence on a given PMH factor $f_{j}$, we suggest using the OWA aggregation operator. The OWA (ordered weighted average) operator is non-compensatory and enables the modelling of different aggregation policies [15]. In particular, we need a conjunctive policy that models a situation of simultaneity. OWA uses a weight $W = \left\{ {\omega_{1} ,\omega_{2} , \ldots } \right\}$ vector to model the character of the aggregation.

Let us define the set $Q$ as the set of questions that satisfy $r_{j} \left( q \right) = 1$, its cardinality being $\left| Q \right|$. Now, the aggregation operator can be formalized with OWA as follows:

2 $$l_{j} \left( c \right) = \mathop \sum \limits_{i = 1}^{\left| Q \right|} \omega_{i} \times s_{\sigma \left( i \right)}^{c} \left( q \right)$$

where $\sigma \left( i \right)$ is a permutation of the scores as $s_{\sigma \left( 1 \right)}^{c} \left( q \right) \ge s_{\sigma \left( 2 \right)}^{c} \left( q \right) \ge \cdots \ge s_{{\sigma \left( {\left| Q \right|} \right)}}^{c} \left( q \right)$.

The set of weights $W$ may be obtained by using a regular monotonic increasing quantifier, as proposed by Yager [16]. A general expression for a RIM is that of Eq. 3. Using this type of quantifier, the weights can be calculated using Eq. 4.

3 $$quant\left( x \right) = x^{\alpha } ,{\text{with}}\quad \alpha \ge 0$$

The parameter $\alpha$ enables to establish the type of merging policy. Three cases can be distinguished:

- For $\alpha = 1$ we get $quant\left( x \right) = x$, which is the unitor quantifier (i.e., mean).
- For $\alpha \to \infty$ we get the universal quantifier (i.e., the conjunctive case: most, all).
- For $\alpha \to 0$ we get the existential quantifier (i.e., the disjunctive case: at least, none).
  4 $$\omega_{i} = quant\left( \frac{i}{n} \right) - quant\left( {\frac{i - 1}{n}} \right)$$

In addition to evaluating the current competence levels of the caregiver, it is important to know which activities can improve each factor. By merging both pieces of information, we will be able to determine which one should be performed first to prevent further deterioration of the caregiver’s mental health (using the method explained in section “The app for PMH improvement at home”).

An impact function has been defined (Eq. 5) to indicate if the PMH factor $f_{j}$ is improved or not when performing the activity $a_{i}$. The values of the *imp* function must be given by the experts who designed the activities.

5 $$imp_{j} \left( {a_{i} } \right) = \left\{ {\begin{array}{*{20}l} { - 1} \hfill & {a_{i} \,does\,not\,improve\,f_{j} } \hfill \\ 1 \hfill & {a_{i} \,improves\,f_{j} } \hfill \\ \end{array} } \right.$$

With the impact values and the PMH factor levels, the following algorithm is proposed to calculate the marginal utility of an activity for each factor and caregiver. The range of the utility score will be $[ - k_{negative} \ldots k_{positive} ]$.
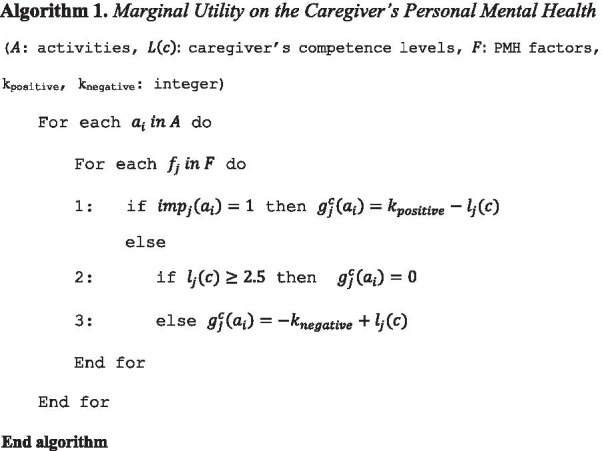


With this algorithm, a negative, zero or positive utility score is assigned to each factor of each activity. In step 1, activities that have a positive impact on the caregivers are considered, assigning a positive utility score that depends on the current level of satisfaction with the corresponding PMH factor. The lower the current level, the higher the utility of the activity. The maximum value will be $k_{positive}$ when the caregiver has a 0 level of achievement. Steps 2 and 3 are for activities with a negative impact, which means that they do not help increase the level of satisfaction with the PMH factor. In this case, if the caregiver has a good enough level (greater than or equal to 2.5), the utility score is set to 0 (neutral case, step 2). However, if the caregiver needs to improve that factor, the utility value will be negative (step 3), up to $- k_{negative}$.

### Ranking of the training activities with MAUT

The most appropriate order of the activities is figured out using the Multi-Attribute Utility Theory (MAUT), based on aggregating the utility score with a mathematical operation that meets certain properties.

To explain the MAUT methodology used in this paper, the following formalization of the ranking problem is proposed:

- A set of $n$ alternatives $A = \left\{ {a_{1} , \ldots ,a_{n} } \right\}$ consisting of the set of training activities available.
- A set of $p$ criteria $G^{c} = \left\{ {g_{1}^{c} , \ldots ,g_{p}^{c} } \right\}$ linked to different factors of caregivers’ positive mental health (PMH). The marginal utility of these criteria are given on a numerical scale from $- k_{negative}$ to $k_{positive}$. When $g_{j}^{c} \left( {a_{i} } \right) > 0,$ it means that the caregiver *c* should do this activity because he/she needs to improve the competence factor represented by $g_{j}^{c}$ and this improvement can be achieved by performing this activity; while having a negative score $g_{j}^{c} \left( {a_{i} } \right) < 0$ means that the user needs to improve the competence on the factor $g_{j}^{c}$, but this activity will not help him/her improve it. A utility score of $g_{j}^{c} \left( {a_{i} } \right) = 0$ means that this criterion $g_{j}^{c}$ is associated to a PMH factor that the user already has. A performance matrix $M = A \times G$ containing the marginal utility scores $g_{j}^{c} \left( {a_{i} } \right)$.

The marginal utility of $g_{j}^{c} \left( {a_{i} } \right)$ must be measured considering the caregiver’s current strong and weak PMH factors, as well as the competences that each training activity is able to improve. The procedure presented in section “Methodology to personalize the ranking of the training exercises” allows to obtain these scores. Once the marginal utility scores have been obtained, the final utility score for each alternative is calculated using the aggregation operator called Sum of Scores (SS) formulated in Eq. 6.

6 $$u^{c} \left( {a_{i} } \right) = \mathop \sum \limits_{j = 1}^{p} g_{j}^{c} \left( {a_{i} } \right)$$

The overall utility scores provide a weak ordering of the alternatives, the higher being the better. A weak ordering $\succcurlyeq$ is a binary relation that is both transitive (i.e. $if \left( {x \succcurlyeq y\,and\,y \succcurlyeq z} \right) \Rightarrow x \succcurlyeq z$) and complete (i.e. $for\,all\,x,y \in A,\,either\,x\succcurlyeq y\,or\,y\succcurlyeq x$). The weak order relation is defined as $x\succcurlyeq y \Leftrightarrow u^{c} \left( x \right) \ge u^{c} \left( y \right).$

The SS method is an additive aggregation operator quite popular due to its simplicity [17]. There are many other aggregator operators in the literature whose mathematical and behavioral properties have already been studied by Torra et al. [18]. The SS operator has the problem of compensation, which means that positive scores are compensated by negative scores. However, this property is appropriate in our paper, because we are trying to measure how the different training activities balance the strong and weak contributions to the achievement of a certain competence. Thus, compensation is not considered a drawback here, but an advantage compared to other methods that try to avoid the compensation of utility values. It is worth noting that the Arithmetic Average (AA) operator will result in the same ranking as SS, as it only normalizes the SS score by dividing it by the number of values ($p$ in this case). It should be noted that AA is a bounded idempotent operator.

When applying additive aggregation operators, the condition of preferential independence must be met. This property states that the marginal utility scores of a criterion $g_{j}$ must be independent of the marginal utility scores of the other criteria. For two criteria, it is formally expressed as:

#### Axiom 1

Preferential independence between two criteria:

For all utility scores $x_{1} ,x_{2} \in g_{i}$ and for all utility scores $y_{1} ,y_{2} \in g_{j}$,

$$\left( {x_{1} ,y_{1} } \right)\succcurlyeq \left( {x_{2} ,y_{1} } \right) \Leftrightarrow \left( {x_{1} ,y_{2} } \right)\succcurlyeq \left( {x_{2} ,y_{2} } \right) and$$

$$\left( {x_{1} ,y_{1} } \right)\succcurlyeq \left( {x_{1} ,y_{2} } \right) \Leftrightarrow \left( {x_{2} ,y_{1} } \right)\succcurlyeq \left( {x_{2} ,y_{2} } \right) .$$

In this paper, the preferential independence of utilities is ensured, because each criterion is associated with one of the different PMH factors, and their marginal utility is assessed separately from the rest of the factors, as explained in the previous subsection.

### Data

#### Participants

A pilot study has been conducted with 111 participants. Initially, a group of 49 non-professional caregivers was selected. Then, to increase the number of people, a group of 62 professionals (i.e., nurses) who were supervising these caregivers were also included in this pilot test of the app. All these users have followed the same intervention plan as if all of them were home caregivers. The supervisors carried out the activities from February 18 to March 19, 2019. The caregivers started on April 8 and finished on May 12, although each individual had exactly 4 weeks to perform the activities. The rest of the paper will analyze these two groups in a unified way, making no distinction between them.

#### Data collection with the questionnaire

The questionnaire used for the evaluation of the caregivers consists of 39 questions, some of which have a positive meaning while others refer to negative aspects [6]. For example, a positive question is: “Q11: I believe I have the ability to put myself in other people’s shoes and understand their answers”. An example of a negative question is: “Q1: I find it difficult to accept other people who have attitudes different from my own”. The answers are given using 4 possible linguistic terms, which are then translated into numerical score as per Table 1.

**Table 1:** Numerical score *s*^*c*^ (*q*) ∈ [0…3] assigned to the type of answers

Answers options	Scores positive question	Scores negative question
Always or almost always	3	0
Quite often	2	1
Sometimes	1	2
Never or almost never	0	3

Each question is related to one of the PMH factors: Personal Satisfaction (F1), Prosocial Attitude (F2), Self-control (F3), Autonomy (F4), Problem-solving and Self-actualization (F5) and Interpersonal Relationship Skills (F6). Table 2 establishes the relationship between each factor and the questions that provide information to assess their level of achievement. For example, question Q1 gives evidence about F2 and question Q11 is related to factor F6.

**Table 2:** Set of questions that satisfy *r*_*j*_ (*q*) = 1 for each factor, defined in Eq. 1

Factor	Survey set of questions
F1	4, 6, 7, 12, 14, 31, 38 and 39
F2	1, 3, 23, 25 and 37
F3	2, 5, 21, 22 and 26
F4	10, 13, 19, 33 and 34
F5	15, 16, 17, 27, 28, 29, 32, 35 and 36
F6	8, 9, 11, 18, 20, 24 and 30

#### Activities design

A team of experts formed by nurses and psychologists defined a set of 20 activities following the decalogue of PMH established by Lluch-Canut [10]. The notation of the activities has the format RxAy, where Rx stands for the x-th recommendation (i.e., the x-th guideline of the protocol) and Ay indicates the y-th activity of this recommendation. For example, activity R1A1 (first activity of the first recommendation) is designed to promote the good things in the caregiver’s life, while R3A2 (second activity of the third recommendation) makes the caregiver think about his/her aspirations in order to be more tolerant and flexible with himself/herself.

There are different types of activities, ranging from simple questions, to mindfulness exercises, to watching videos, among others. All of them are aimed at improving some aspects of PMH. The relationship between the activities and the factors they seek to improve was established by experts, and shown in Table 3. One activity usually influences more than one factor at the same time.

**Table 3:** Set of activities that improve each factor of PMH

Factor	Activities that improve each factor
F1	R1A1, R2A1, R2A2, R5A1, R5A2, R6A3, R9A1 and R9A3
F2	R2A1, R2A2, R3A1, R6A2, R8A1, R8A2 and R9A2
F3	R1A2, R3A2, R4A1, R4A2, R7A1 and R7A2
F4	R1A1, R3A2, R5A1, R5A2, R6A1, R6A2, R6A3, R7A1 and R7A2
F5	R1A2, R4A1, R4A2, R8A1, R8A2, R9A1 and R9A2
F6	R3A1, R6A1, R9A2 and R9A3

#### Activities sample

To test the methods proposed in section “Methods”, a subset of 7 activities was selected. According to the notation given in Eq. 5, each activity can be described with a vector with the impact on each factor $imp_{j}$ expressed with the values 1 and -1. In Table 4, the selected subset of 7 activities is formalized using this vector $imp\left( {a_{i} } \right).$ A positive number indicates that the activity is designed to improve this factor (f.i. R9A3 and F1), while a negative number indicates that the activity does not affect the satisfaction of the corresponding factor (e.g.,. R9A3 and F2).

**Table 4:** Values of the *imp* function defined in Eq. 5 for a subset of activities *a*_*i*_ and each factor *f*_*j*_

Activity	F1	F2	F3	F4	F5	F6
R1A1	1	− 1	− 1	1	− 1	− 1
R1A2	− 1	− 1	1	− 1	1	− 1
R2A1	1	1	− 1	− 1	− 1	− 1
R6A2	− 1	1	− 1	1	− 1	− 1
R7A1	− 1	− 1	1	1	− 1	− 1
R8A1	− 1	1	− 1	− 1	1	− 1
R9A3	1	− 1	− 1	− 1	− 1	1

## Results

The experiments have been conducted in the pilot test with the above-described data. This section is divided into two subsections. In the first one, we study the behavior of the OWA operator for the different aggregation policies, which depend on the value used to generate the set of weights. In the second one, we present the results of the calculation of the marginal utility scores with OWA and the ordering of the sample of activities using MAUT on a subset that is representative of the caregivers.

### Results of the aggregation with OWA to determine the PMH level of each caregiver

**Fig. 3:**
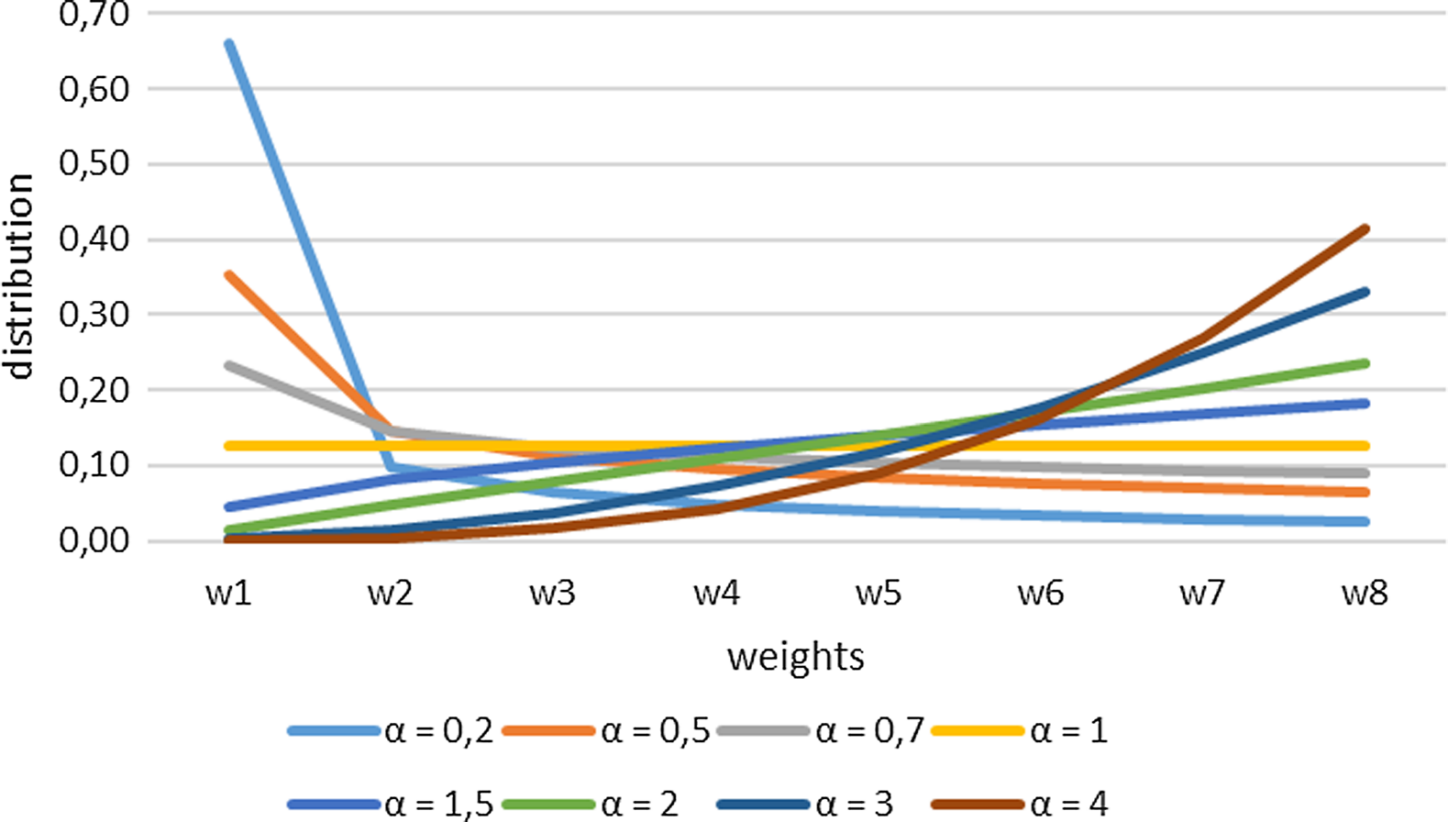
Values of the ordered weighted average, OWA, weights for different $\alpha$

Following the methodology proposed in section “Methodology to personalize the ranking of the training exercises”, the OWA operator has been used to aggregate the scores obtained in the questionnaire. This operator (Eq. 2) needs a set of weights $W = \left( {\omega_{1} ,\omega_{2} , \ldots ,\omega_{Q} } \right)$ that defines the aggregation policy, from conjunctive (i.e., simultaneity is required) to disjunctive (i.e., substitutivity/compensation is applied). In this paper, the weights are generated using the quantifier function Eq. 4, which depends on the parameter $\alpha$. Different values of this parameter have been tested to compare how the weights are distributed in the vector, in particular $\alpha = 0.2, 0.5, 0.7, 1, 1.5, 2, 3\,{\text{and}}\,4$. The length of this vector $W$ depends on the number of questions related to each factor, which is between 5 and 9 (see Table 2). Figure 3 represents the values of *W* for the 8 answers obtained for factor F1. With $\alpha = 0.2$, the weight is concentrated on the highest value (i.e., the first one after the descending ordering), with $\omega_{1} = 0.66$; while the rest quickly decrease to almost 0. On the opposite side, with $\alpha = 4$ the highest weight goes to the lowest score, the four highest values being practically ignored. Between these two polarized cases, the others make a more balanced distribution of the weights. As expected by definition, when $\alpha = 1,$ we have the arithmetic average.

In a second experiment, a subset of 12 caregivers with different needs was selected for testing the method proposed in this paper. The code assigned to each caregiver is the same one that will be used throughout the presentation of results, and for this reason in this first table it does not have consecutive values. We first applied all these 8 different policies to the answers of all caregivers. An illustrative example is given in Tables 5 and 6 for a subset of 5 users. Table 5 shows the scores for the 8 questions related to factor F1. Table 6 shows the aggregated score to indicate the PMH level for factor F1, obtained with different values of the parameter $\alpha$.

**Table 5:** Scores of the answers provided by 5 users to questions related to F1. The id of the question is formed by “*q*” plus the question number (see Table 2)

Caregiver id	*s*^*c*^ (*q*4)	*s*^*c*^ (*q*6)	*s*^*c*^ (*q*7)	*s*^*c*^ (*q*12)	*s*^*c*^ (*q*14)	*s*^*c*^ (*q*31)	*s*^*c*^ (*q*38)	*s*^*c*^ (*q*39)
User 1	3	2	3	3	3	3	0	2
User 4	3	2	3	2	2	2	2	2
User 7	1	1	0	1	2	2	0	0
User 10	0	1	2	2	1	2	0	0
User 12	3	2	3	3	3	3	3	3

**Table 6:** Results of OWA aggregation for different values of α

Caregiver id	α = 0.2	α = 0.5	α = 0.7	α = 1	α = 1.5	α = 2	α = 3	α = 4
User 1	2.86	2.66	2.54	2.38	2.13	1.92	1.58	1.32
User 4	2.76	2.50	2.38	2.25	2.13	2.06	2.02	2.00
User 7	1.67	1.29	1.10	0.88	0.62	0.45	0.26	0.16
User 10	1.73	1.40	1.22	1.00	0.72	0.53	0.30	0.17
User 12	2.97	2.94	2.91	2.88	2.82	2.77	2.67	2.59

We can observe that some users have higher scores in this factor, such as user4 and user12; others provided answers with lower scores, such as user7 and user10. The case of user1 is particularly interesting because it seems to meet most aspects of factor F1, but not the one related to question *q*38. The aggregated result should detect this case and give a lower score level than the ones of user4 and user12. Table 6 shows that not all parameter values allow this distinction to be made. The most suitable results are the ones of $\alpha = 3$ and $\alpha = 4$, which correspond to the conjunctive case.

**Fig. 4:**
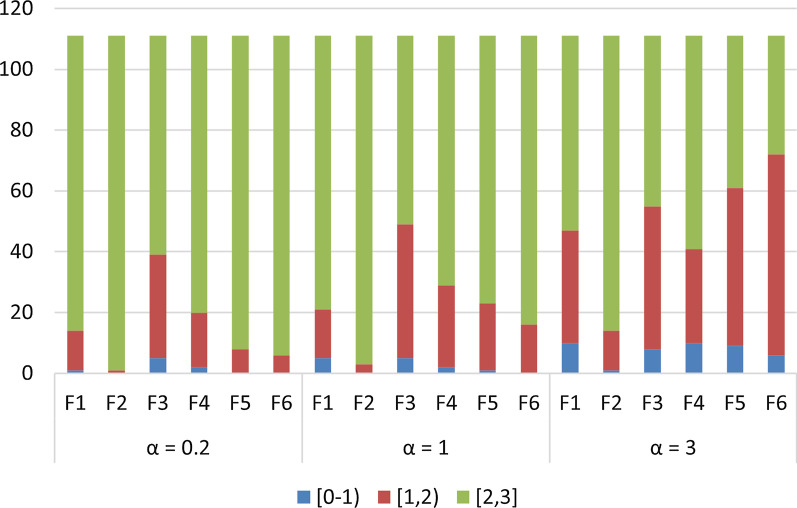
Histogram of the PMH level obtained for different values of $\alpha$. F1, Personal Satisfaction; F2, Prosocial Attitude; F3, Self-control; F4, Autonomy; F5, Problem-solving and Self-actualization and F6, Interpersonal Relationship Skills

Additionally, a study of the distribution of the overall PMH satisfaction level on factors $l_{j} \left( c \right)$ was carried out for all participants. 3 intervals [0, 1), [1, 2) and [2, 3] were considered for each factor. Figure 4 shows the histogram, in which we can observe that only with the conjunctive policy ($\alpha = 3$) we can properly distinguish the 3 intervals. With the neutral or optimistic approach, most users end up with a level higher than 2.

**Fig. 5:**
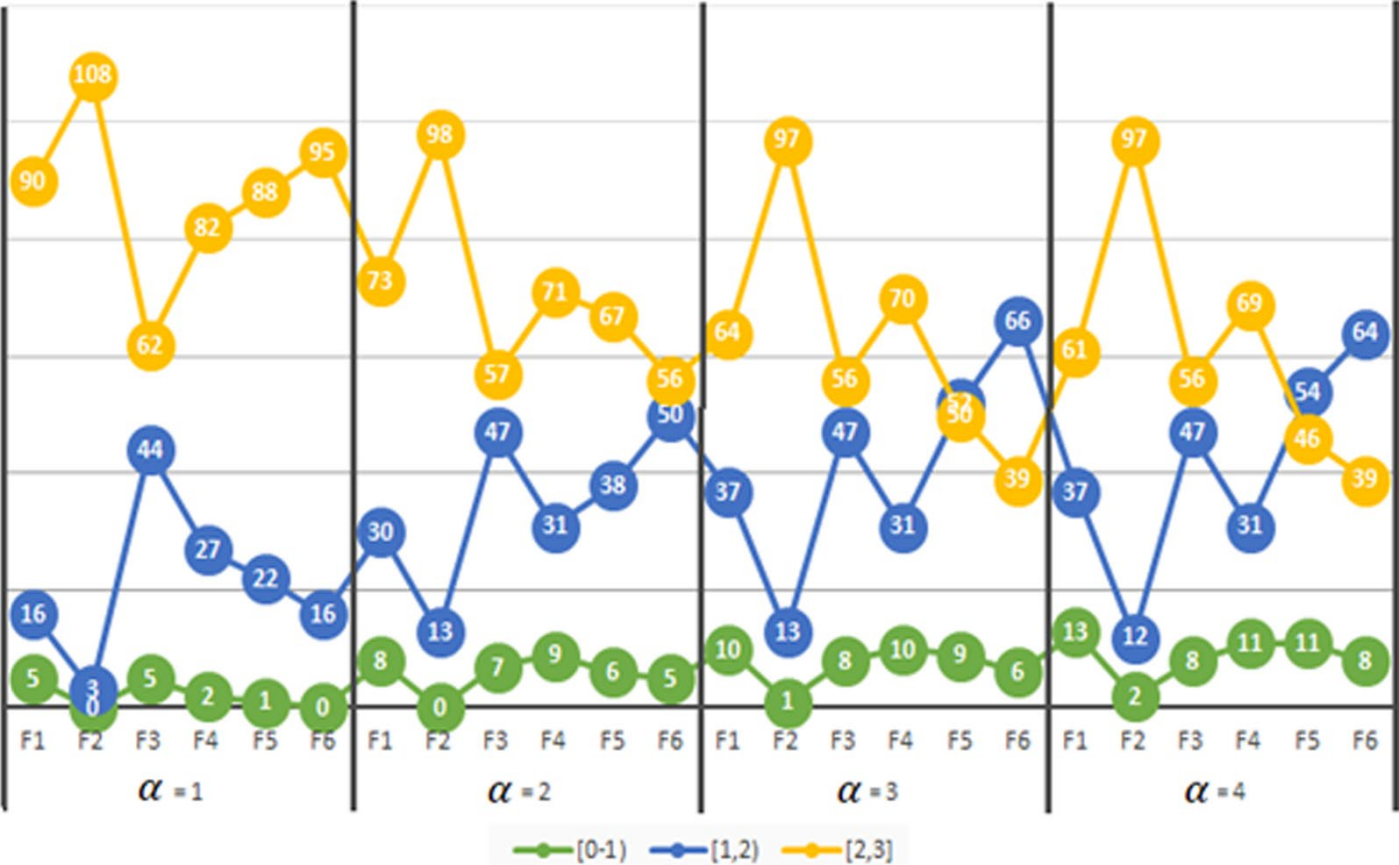
Distribution of the user’s PMH values calculated with conjunctive aggregation policies, in comparison with the neutral one

A complementary chart is shown in Fig. 5, which displays the number of participants in each level interval for each factor and for different values of the parameter, starting from the neutral one and then with 3 levels of conjunctiveness. While $\alpha = 2$ starts to decrease the number of caregivers in the third interval (yellow), it is with $\alpha = 3$ that we have the best distribution (almost equal to $\alpha = 4$). Notice, for example, that in factor F1, 10 caregivers have a PMH level in the range [0–1), 37 caregivers are in range [1–2) and 64 in the highest range [2, 3]. A quite different distribution is found with $\alpha = 1$, where the majority of caregivers have a value in the range [2, 3]. Therefore $\alpha = 3$ is chosen as the best value to generate the OWA weights in all factors. So, the best aggregation policy is the conjunctive one, which requires jointly satisfying a subset of the questions in order to assign a high level to a PMH factor.

### Results of the calculation of utility scores and ranking of activities

To test the methods proposed in sections “Calculation of the marginal utility scores for a given caregiver” and “Ranking of the training activities with MAUT” for rating and ranking the set of activities, we will use a subset of 12 caregivers and 7 exercises. The input data needed is as follows:

The caregiver’s PMH level score on each factor, obtained with the use of the OWA operator with conjunctive weights ($\alpha = 3$). The 12 selected caregivers’ values are shown in Table 7.

**Table 7:** Values indicating the PMH level of the 12 caregivers for every factor

Caregiver id	F1	F2	F3	F4	F5	F6
User 1	1.58	2.51	2.22	1.06	0.88	1.82
User 2	2.05	2.51	1.07	1.07	1.79	2.63
User 3	1.76	3.00	3.00	1.54	3.00	1.36
User 4	2.02	2.51	2.06	2.01	2.47	2.63
User 5	2.42	3.00	1.22	2.51	1.88	3.00
User 6	3.00	1.58	2.51	3.00	3.00	1.36
User 7	0.26	1.24	0.58	0.58	0.34	1.65
User 8	3.00	3.00	2.51	3.00	2.70	1.71
User 9	2.42	2.22	3.00	2.51	3.00	1.39
User 10	0.30	3.00	1.22	0.28	0.21	0.57
User 11	1.26	1.58	0.00	0.52	0.71	0.71
User 12	2.67	1.58	2.06	2.51	2.30	2.00

The impacts of the activities in the 6 factors, presented in Table 4.

Using these two inputs, the proposed method (Algorithm 1) is applied to calculate the marginal utility scores for each PMH factor. This algorithm weights the PMH levels using the degree of impact of each activity on each factor. The algorithm uses two constants that establish how the initial PMH values are modified depending on whether the impact exists or not: k_positive_ (for reward) and k_negative_ (for penalty). After an experimental analysis, the values of k_positive_ = 4 and k_negative_ = 3 have been used.

To illustrate the application of this algorithm, let us take the caregiver with identifier user3, who has an excellent level in factors F2, F3 and F5 and a medium–low level in factors F1, F4 and F6. The marginal utility scores obtained for this caregiver are shown in Table 8.

**Table 8:** Marginal utility scores for User 3 in a subset of activities

Activity id	F1	F2	F3	F4	F5	F6
R1A1	2.24	0.00	0.00	2.46	0.00	− 1.64
R1A2	− 1.24	0.00	1.00	− 1.46	1.00	− 1.64
R2A1	2.24	1.00	0.00	− 1.46	0.00	− 1.64
R6A2	− 1.24	1.00	0.00	2.46	0.00	− 1.64
R7A1	− 1.24	0.00	1.00	2.46	0.00	− 1.64
R8A1	− 1.24	1.00	0.00	− 1.46	1.00	− 1.64
R9A3	2.24	0.00	0.00	− 1.46	0.00	2.64

The same algorithm has been applied to the rest of the caregivers. Subsequently, the sum of scores method has been used to aggregate all marginal utility scores and obtain the final overall utility value of each activity for each user (see Table 9). In this table, the values in italics indicate that the activity will help the caregiver to improve some of the factors he/she needs to improve. Following the example of user3, the best activities are R1A1 (which improves factors F1 and F4) and R9A3 (which improves factors F1 and F6), which were his weak points. On the other hand, activities R1A2 and R8A1 can be performed later, since they are focused on factors that the user already satisfies.

**Table 9:** Overall utility scores for each activity and users

Caregiver id	R1A1	R1A2	R2A1	R6A2	R7A1	R8A1	R9A3
User 1	*1.26*	*0.37*	− 2.12	− 1.08	*0.00*	− 0.71	− 0.24
User 2	*1.74*	*2.26*	− 1.63	*0.33*	*3.70*	− 1.11	− 1.75
User 3	*3.06*	− 2.34	*0.13*	*0.58*	*0.58*	− 2.34	*3.42*
User 4	*2.51*	*1.49*	*1.01*	*1.03*	*2.41*	*0.11*	*0.90*
User 5	*0.16*	*4.33*	− 0.33	− 1.00	*2.57*	*0.76*	− 0.33
User 6	− 1.07	− 0.58	*1.78*	*1.78*	− 0.58	*1.78*	*2.22*
User 7	− 1.03	− 1.18	− 2.34	− 2.99	− 1.66	− 2.51	− 3.17
User 8	*0.71*	*1.49*	*0.71*	*0.71*	*1.20*	*1.01*	*3.29*
User 9	*0.67*	− 0.97	*1.75*	*1.08*	− 0.49	*0.59*	*3.41*
User 10	*0.42*	− 1.27	− 5.02	− 4.98	− 1.42	− 4.84	− 0.17
User 11	− 2.78	− 0.65	− 4.89	− 3.41	− 0.26	− 3.80	− 3.16
User 12	− 1.24	*1.22*	*1.12*	*1.28*	*0.30*	*2.19*	*0.26*

The final step involves using these overall utility scores to rank the activities. The results for these 12 caregivers are shown in Table 10. We can again observe that the first activity for user3 will be R9A3, followed by R1A1 (as explained before). For some other users, we can also check the appropriateness of the exercise of day 1 and day 2. User11 has a clear lack of factor F3, and the activities that focus on this factor are R7A1 and R9A3, which are proposed to him/her for the first two days. Similarly, activity R1A2 is the best for user5, as it focuses in F3 and F5, which are this user’s lowest scores.

**Table 10:** Procedure for ranking the activities of the caregivers

	Order relation among the selected activities							Ranking of the selected training activities						
Selected users	R1A1	R1A2	R2A1	R6A2	R7A1	R8A1	R9A3	1	2	3	4	5	6	7
User 1	1	2	7	6	3	5	4	R1A1	R1A2	R7A1	R9A3	R8A1	R6A2	R2A1
User 2	3	2	6	4	1	5	7	R7A1	R1A2	R1A1	R6A2	R8A1	R2A1	R9A3
User 3	2	7	5	4	5	3	1	R9A3	R1A1	R7A1	R6A2	R2A1	R8A1	R1A2
User 4	1	3	5	4	2	7	6	R1A1	R7A1	R1A2	R6A2	R2A1	R9A3	R8A1
User 5	4	1	6	7	2	3	5	R1A2	R7A1	R8A1	R1A1	R9A3	R2A1	R6A2
User 6	7	6	4	3	5	2	1	R9A3	R8A1	R6A2	R2A1	R7A1	R1A2	R1A1
User 7	1	2	4	6	3	5	7	R1A1	R1A2	R7A1	R2A1	R8A1	R6A2	R9A3
User 8	7	2	6	5	3	4	1	R9A3	R1A2	R7A1	R8A1	R6A2	R2A1	R1A1
User 9	4	7	2	3	6	5	1	R9A3	R2A1	R6A2	R1A1	R8A1	R7A1	R1A2
User 10	1	3	7	6	4	5	2	R1A1	R9A3	R1A2	R7A1	R8A1	R6A2	R2A1
User 11	3	2	7	5	1	6	4	R7A1	RUA2	R1A1	R9A3	R6A2	R8A1	R2A1
User 12	7	3	4	2	5	1	6	R8A1	R6A2	R1A2	R2A1	R7A1	R9A3	R1A1

## Discussion

The framework of this study is a research project aimed at developing a new mHealth technology to promote positive mental health for non-professional caregivers. In previous works [12], we presented the design and validation protocol for an app developed to promote positive mental health to non-professional home caregivers. Health interventions using mobile technologies to improve patient care at home are well known, and their effectiveness has been proven by several studies [19, 20].

However, the first version of the app proposed the same set of activities to all users, following a predefined and fixed order. This approach was considered insufficient to provide a real and effective response to the users’ needs, so the goal of this paper was to find new methods to personalize the app. This hypothesis is also found in other papers in the literature and some attempts have been made to personalize computer tools. Some authors take into account the needs of the caregiver, but mainly in terms of improving the communication with health professionals when support is needed for the care of the elderly [21, 22]. Fewer papers focus on the caregiver’s wellbeing. McKechnie et al. [23] evaluated a total of 14 empirical studies of computer-mediated interventions with favorable user acceptance. Wasilewski et al. [24] reviewed 53 web-based systems, but less than half included tools for managing caregiver stress. These authors and some others, Sherifali et al. and Cristancho-Lacroix et al. [20, 25, 26], stress that more dynamic, personalized and social interventions need to be developed instead of traditional web-based interventions. In this regard, this paper contributes to the automatic personalization of an intervention plan to improve the caregivers’ mental health according to each person’s needs.

In the framework of home care assistance, the burden of care can adversely affect the health of the caregiver, as explained in the introduction of this paper. For this reason, the methods presented in this paper may help improve the conditions of each caregiver in a different way, because they allow for personalizing an intervention plan based on each person’s needs.

The methodology for generating a personal ranking of a set of activities is divided into two steps. In the first step, the OWA operator is used to calculate the utility of each activity for each factor of PMH and for a given caregiver. The OWA can be configured to do different kinds of aggregation of input scores. Bearing in mind that we want to detect needs and strengths of the caregivers, the aggregator should not compensate for low scores in the answers but focus on detecting them. The results shown in section “Results of the calculation of utility scores and ranking of activities” confirm that the aggregation should be conjunctive, and only if all the answer scores are high can the factor be considered as satisfied. Algorithm 1 is then used to calculate how much an activity impacts on a particular factor and what utility or improvement can the caregiver expect on this PMH factor. After an experimental analysis, the values of k_positive_ = 4 and k_negative_ = 3 have been identified as the best ones. Therefore, the conclusion is that the reward for activities that improve the level of a certain PMH factor should be greater than the penalty for activities that do not improve this factor.

In the second step of the methodology, the sum of scores operator is used to calculate an overall utility for each activity, considering the factors the activity focuses on. The results have shown that each person receives a personalized ranking of the activities, which optimizes the satisfaction of the lowest PMH levels as soon as possible. Despite being a simple mathematical operator, the sum of the scores is adequate to detect the activities that will have a greater impact on the caregiver and will help him/her feel better in the hard work of home care assistance.

Although this methodology was tested and the most appropriate values for the parameters were established, the quantity of data was not very large. Additional testing would be interesting to confirm that the equations and algorithms proposed are robust and useful to many caregivers. Moreover, the fact that the study included both professional and non-professional caregivers may have some influence on the results. We plan to conduct a separate analysis of the behavior of this proposed method in both groups separately and compare the opinions expressed by each of them.

The limitation of the proposed methodology is that it is not able to select a subset of the activities for each caregiver. If the experts defined more PMH factor-related activities to be included in the app, a new step could be added to find the best subset for each user. The solution may not be to take the subset of activities at the top of the ranking, because they may be too similar and focus only on some of the factors that need to be improved. More complex decision-making support techniques to identify groups of suitable activities should be studied to deal with this limitation.

It is worth noting, as final point of discussion, that the proposed methodology can be easily adapted to similar problems. The technical details given and the formalization proposed intend to facilitate this adaptation. For example, it may be useful to personalize a plan of yoga or pilates exercises at home, to recommend an ordered set of rehabilitation exercises or even to build an individual training plan to reduce weight. In the current unprecedented situation of the COVID-19 pandemics, having tools to improve people’s health with smartphone apps has become extremely important. This paper is aimed to make a small contribution in this direction.

## Conclusion

Nowadays, mhealth is a trending topic, and several apps are being developed to help in different healthcare frameworks. An interesting field is the one that assists the caregivers’ mental health. However, most systems are static apps, serving as self-help books. In this paper, a new method is proposed to enable the construction of a personalized smartphone app that considers the needs of each user.

Dynamic, flexible, personalized and easy-to-use were the essential design features we had in mind when developing the methodology, which is based on a combination of mathematical aggregation operators and multi-criteria utility techniques. The methods are formalized and presented in detail in this paper, and they are divided into two main stages: evaluating the users’ needs, and rating and ranking the impact of the activities.

The paper explains the use of an app that proposes a set of 20 home exercises to non-professional caregivers. The results of a pilot test have been used to fix the values of some parameters of the methods, while showing the flexibility in the configuration of the proposed techniques.

Now it is necessary for this work to take a step forward and prove its effectiveness with a clinical trial [12]. Promising results are expected for the methodology presented in this paper as a useful tool to prevent caregiver burden. Furthermore, this process of creating personalized apps should be an example of good practice in mhealth development.

## Data Availability

The datasets during and/or analysed during the current study available from the corresponding author on reasonable request.

## Abbreviations

PMH: Positive mental health
OWA: Ordered weighted average
MAUT: Multi-attribute utility theory
